# Gully evolution and geomorphic adjustments of badlands to reforestation

**DOI:** 10.1038/srep45027

**Published:** 2017-03-22

**Authors:** J. A. Ballesteros Cánovas, M. Stoffel, J. F. Martín-Duque, C. Corona, A. Lucía, J. M. Bodoque, D. R. Montgomery

**Affiliations:** 1 Climatic Change and Climate Impacts, Institute for Environmental Sciences, University of Geneva, Boulevard Carl-Vogt 66, CH-1205 Geneva, Switzerland; 2 Department of Earth and Environmental Sciences, University of Geneva, CH-1205 Geneva, Switzerland; 3 Department of Geodynamics, Complutense University of Madrid (UCM) and IGEO (CSIC, UCM), E-28040, Madrid, Spain; 4 Centre National de Recherche Scientifique (CNRS) UMR6042 Geolab, F-63006, Clermont-Ferrand Cedex, France; 5 Faculty of Science and Technology, Free University of Bozen-Bolzano, I- 39100, Bolzano, Italy; 6 Centre of Applied Geosciences, Eberhard Karls University of Tübingen, G- 72074, Tübingen, Germany; 7 Department of Geological and Mining Engineering, University of Castilla-La Mancha (UCLM), Avda. Carlos III s/n, 45071 Toledo, Spain; 8 Department of Earth and Space Sciences. University of Washington, WA 98195, Seattle, USA

## Abstract

Badlands and gullied areas are among those geomorphic environments with the highest erosion rates worldwide. Nevertheless, records of their evolution and their relations with anthropogenic land transformation are scarcer. Here we combine historical data with aerial photographs and tree-ring records to reconstruct the evolution of a badland in a Mediterranean environment of Central Spain. Historical sources suggest an anthropogenic origin of this badland landscape, caused by intense quarrying activities during the 18^th^ century. Aerial photographs allowed detection of dramatic geomorphic changes and the evolution of an emerging vegetation cover since the 1960s, due to widespread reforestation. Finally, tree-ring analyses of exposed roots allowed quantification of recent channel incision of the main gully, and sheet erosion processes. Our results suggest that reforestation practices have influenced the initiation of an episode of incision in the main channel in the 1980s, through the hypothesized creation of disequilibrium in water-sediment balance following decoupling of hillslopes from channel processes. These findings imply an asymmetry in the geomorphic response of badlands to erosion such that in the early evolution stages, vegetation removal results in gullying, but that reforestation alone does not necessarily stabilize the landforms and may even promote renewed incision.

Badlands and gullied areas have some of the highest erosion rates worldwide^1^. Nevertheless, records of their historical and recent evolution are scarce and often limited to comparisons of presumed initial conditions and the known present state^2^. Badland morphologies typically develop in horizontally stratified and relatively impermeable lithologies (e.g., marls), although they also form on poorly consolidated sands. Areas prone to badland formation cover a wide range of climatic zones, but are typically found in semi-arid environments with marked seasonal contrasts and to a lesser extent in sub-humid and humid regions^1^,^3^,^4^. Gully formation is usually related to changes in the base level^5^ and alteration of vegetation cover on soils developed on highly erodible geological material^6^. This alteration may be induced by climatic changes and the alternating occurrence of severe droughts and extreme rainfall^6^. Inappropriate agricultural practices, overgrazing, logging, quarrying or dumping of spoil heap deposits may result in alteration of the vegetation cover and thereby enhance erosion^7^,^8^. Because of such actions, anthropogenic activities are the main geomorphic agent of current land degradation leading to gullying^9^.

As a consequence, many landforms and landscapes over large areas of Earth’s surface, especially in the Old World, cannot be understood without knowledge of land surface transformation by human activities^9^,^10^. Mining, plowing, or infrastructure expansion has moved huge amounts of earth and led to accelerated erosion. The scars of old mining activities can evolve to gullied landscapes^11^ such that eroded sediments end up as colluvium on hillslopes, as alluvial cones on piedmonts, and as alluvium in floodplains, in ways that shape what we see now and interpret in geomorphic terms. Human activities involving earth movement can act as a main landscape disturbance factor, comparable to the most obvious ones (e.g., tectonics or climate shifts), and shift the geomorphic system toward new landscape equilibria^12^.

In the context of global change, the understanding of past and present human-induced landscape change is essential to inform proper land-use management and to assure the provision of ecosystem services for sustainable use of land, soil, and water, or for ecological restoration^13^,^14^,^15^,^16^. In badland systems, long-term observations are usually unavailable or restricted to aerial photograph interpretation, which limits interpretation of human-induced, geomorphic change^17^. Here, we use an unusually dense record and outstanding example of erosion processes in a Mediterranean environment (Central Spain) to investigate gullying processes on unique silica sand slopes of a set of mesas thought to have been triggered by quarrying activities^17^,^18^,^19^. The development of gullying on silica sand slopes is characterized by a low stability over geomorphologic timescales^7^,^20^, and the gullied slopes are representative of many Mediterranean landscapes. Intense erosion rates in this region caused frequent sedimentation on roads and agricultural lands and resulted in reforestation initiatives in the second half of the 20^th^ century^17^.

The gully evolution analyzed here provides an example of a larger setting of landscapes which originate in the history of humans modifying the land in the area^19^. We applied a multi-proxy approach based on historical archives, long-term aerial photography and tree-ring analysis of exposed roots to interpret the origin of this landscape and to understand geomorphic adjustments to historical land-use changes. Based on the gathered data, we conclude that land-use changes have affected and still are affecting erosional processes in this badland landscape, but not necessarily as one might hypothesize. Indeed, reforestation had controlled erosion on the slopes but appears to have triggered renewed gullying in the channel, generating undesired geomorphic adjustments in the system. This example illustrates how the connectivity between hillslope and channel processes acting in a system may be crucial in terms of geomorphic restoration or reclamation of disturbed lands^13^,^14^,^15^,^16^, and could play a key role in achieving suitable restoration and management actions.

## Gullied Landscape

**Figure 1:**
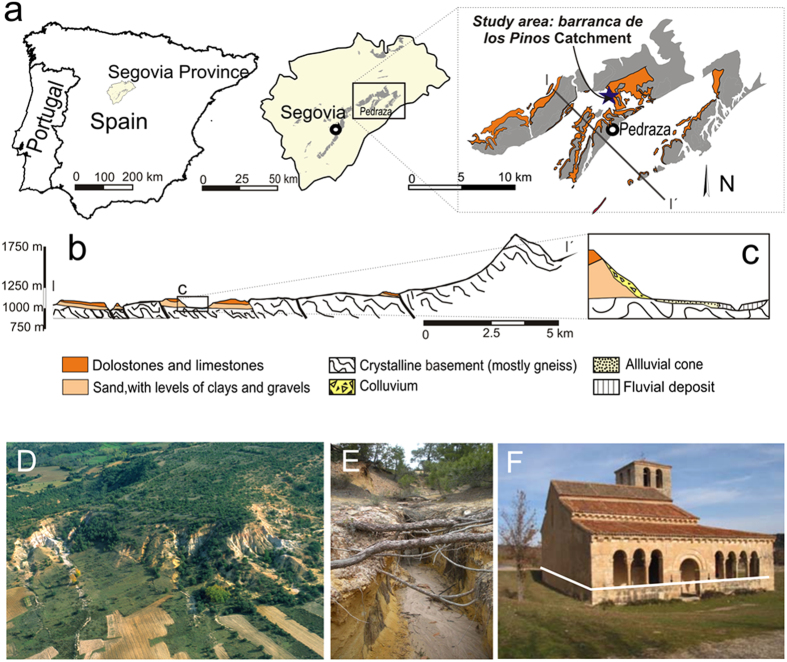
Location of the study area in a geographical (**a**) and in a geological context (**b**). (**c**) The crystalline basement occurs beneath alluvium in the valley bottom, and shows no evidence of re-adjustment due to changes in base level. (**d**) Aerial view of the studied gullies. (**e**) Exposed roots in the channel analyzed in this paper. (**f**) Example of recent (historic) geomorphic activity in the study area. The white line represents the level to which this medieval church was buried, due to continued issuance of sediments from an alluvial cone fed by gullies developed on silica sand slopes. Maps have been created using ArcGIS 10.1 (www.esri.com) and CorelDrawX7 (www.coreldraw.com).

The badland area is located in the Spanish Central System, Province of Segovia (41° 9′ 30′′N; 3° 48′ 30′′ W) at an elevation of 1065 m (Fig. 1). Analyses were performed in a representative badland with a gully system, 1.32 ha in size and with slopes gradients >30% on more than one-third of the site. The area is characterized by a set of gullies developed on sand slopes which are interpreted to be the result of geomorphic evolution of former quarries. The site is underlain by Upper Cretaceous marine (limestone and dolostone) and fluvial sediments (gravelly clayey and silica sand). These sediments were deposited on a basement of erosion-resistant Proterozoic and Paleozoic metamorphic and igneous rocks of the Hercynian massif, which outcrop in the Cega River along the main valley bottom of the area, and which promote long-term stability of the channel profile. The region is characterized by temperate dry and warm summer (Csb type), with an average annual temperature of 11.4 °C and average annual precipitation of 680 mm (range: 443–992 mm), generally concentrated during winter-early spring months, with maximum daily intensity of 120 mm recorded at the study site in 1982^18^. From May 2007 to December 2011, a rain gauge station was installed in the gully, which recorded heavy rainfalls in September 2008, with a maximum daily rainfall of 69.4 mm and a maximum 30-min intensity of 72.4 mm h^−1^. Since the mid-twentieth century, the area experienced general abandonment of rural activities and mining (i.e., reduced extraction of sand in the gullies), which together with reforestation activities have favored forest recovery at the site (see Supplementary Figs S1 and S2).

## Results and Discussion

### The origin of the gullied landscape

Historical archives establish that the study area has been subject to intense human activity since Medieval times (see Supplementary Table S3 and Fig. S4). Documentary sources suggest that landscape modification in this Mediterranean environment may even have started during the Roman period. Limestone and dolostone quarrying in the caprock of the mesas and cuestas where gullies now exist were quite extensive after the medieval period, in particular for the provision of building material during the establishment of nearby settlements, for houses, churches and stone-field fences. Pollen analyses corroborate that during the last millennium the region suffered intense deforestation^21^, motivated by the intrinsic economic value of the forest^22^,^23^, and demand for grazing and agricultural lands. Subsequent landscape transformation due to human activities was more intense during the 18^th^ century than it is today^19^.

Despite the well-documented historical socio-economic activity in the region, records of sand quarrying or the description of related geomorphic features cannot be found for during the Medieval period. To our knowledge, the first historical description about the existence of sand quarries only goes back to 1864 CE (see Supplementary Table S3). These findings on sand quarrying match with known land-use changes in the area, and it could be favored by the needs of the Spanish glass industry during the period of the Bourbon dynasty. Moreover, physically-based evidence of intense sedimentation burying a Romanic church is located in the alluvial cone of a nearby gully, with up to 105 cm of a sandy deposit produced from badlands until restoration works at the religious building were carried out in the 1970s. Since documentary sources demonstrate that the church was first restored in 1756 CE and operated as an important ecclesiastic site during the 18^th^ century, the intense sedimentation occurred in conjunction with the abandonment of the church during depopulation of the region in the 19^th^ century.

In recent times, aerial photo interpretation confirms the existence of mining activities before the mid-20^th^ century. We have evidence for the existence of different sectors of systematic sand exploitation and collection and a network of small extraction paths close to the catchment in the aerial photography of 1946. This mining activity is, however, absent in the aerial photos since 1956 as well as in historical records, and it’s cessation is further confirmed by local testimonies. The analysis of sequential aerial photographs suggests that the gullied slope area increased by up to 66% between 1946 and 2016, defining an average gully head retreat of almost 0.18 ± 0.09 m yr^−1^. During this period, detailed analyses of the geomorphic features inside the gullied slope (badland) suggest ‘cut-and-fill’ dynamics and enhanced erosion that transferred and deposited large amounts of sediment farther downstream. These processes led to important geomorphic changes and badland evolution. In particular, we detected large changes in the longitudinal profile of the main gully stem of the drainage network. The first available set of aerial photos taken in 1946 shows the studied gully disconnected from the neighbouring gully systems, and evacuating sediment directly to its fan (see Supplementary Fig. S5). In contrast, the aerial photos of 1956 and subsequent years show a connection between the drained network of the studied gully and nearby gullies. Since the late 1970’s, aerial photography shows evidence of reforestation activities and the colonization of previously bare surfaces by *Pinus pinaster* Ait. trees. The tree-covered area within the studied gully increased by up to ~70% since 1979.

The core of the above body of evidence reinforces the hypothesis of the anthropogenic origin of this typical Mediterranean landscape, and reveals that active erosion-sedimentation processes have been central to the evolution of this landscape in recent centuries. Despite the fact that sporadic limestone and dolostone quarrying activities started in the region during medieval times (and possibly even during Roman times), the lack of historical description of mining activity in the area suggests that systematic sand mining activity started much later, likely after the 18^th^ century. Accounting for the long-term channel stability in the valley bottom, these observations support the hypothesis that quarrying activities significantly modified hillslope gradients. We argue that anthropogenic activities in the region triggered intense erosion and sedimentation processes. This argument is supported by recent measurements of active processes in this catchment, where sediment transport rates have been found to be extremely high relative to other badlands areas, but where sediment delivery ratios are low (23%)^18^, suggesting low hillslope-channel connectivity and consequently a high sensitivity to geomorphic changes, characteristic of early stages of badland system evolution^24^.

### Multi-decadal erosion rate reconstruction

Exposed roots have allowed quantification of erosion processes within the badland with annual resolution over recent decades^25^,^26^. Average estimated sheet erosion in three homogenous response units (HRU) was 6.7 ± 3.8 mm yr^−1^, with values ranging between 1.6 and 18.7 mm yr^−1^ (n° = 75). These rates are comparable to those recorded in many of the most erosive hotspot sites worldwide^27^. Observed sheet erosion rates at the site are highly dependent of HRU characteristics, regardless of the average slope angle. Sheet erosion in moderate (~14 ± 5°) sand slopes was larger (8.1 ± 3.8 mm yr^−1^) than in steeper (~26 ± 4°) slopes where sand was covered by vegetative litter (5.5 ± 3.3 mm yr^−1^), but similar to values observed on bare sandy interfluves (7.3 ± 4.1 mm yr^−1^) with moderate slopes (~16 ± 5°). Differences between HRUs clearly point to the protective effect of increasing tree cover in recent years, due to the positive role of vegetation in reducing splash and sheet erosion^28^,^29^. In addition, short-term (2008–2011) monitoring revealed lower erosion rates (at least by half) in HRUs characterized by the presence of a pine-needle litter as compared to those with uncovered or bare surfaces^26^.

**Figure 2:**
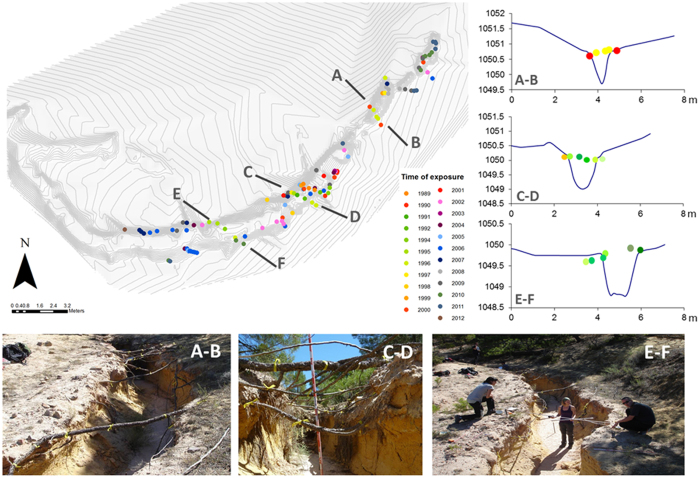
Distribution of the exposed roots analyzed along the channel, as well as three examples of the evolution of different profiles characterized by exposed roots. Map has been created using ArcGIS 10.1 (www.esri.com).

Tree-ring records also indicate that the main channel of the badland catchment has experienced fast incision to form a “valley floor gully” *(arroyo)* with vertical slopes and a knickpoint between 1989–2015, with peaks in incision and related gully widening in 2006, and further major erosion events in 1996, 2000, 2002, 2007, 2009, 2010, and 2011 (Fig. 2). Despite the absence of exposed roots in the lowermost gully section, our findings show that incision at the site started in the late 1980s, with an average annual knickpoint retreat rate of 0.63 ± 0.68 m yr^−1^ since at least 1989, gully incision of 0.09 ± 0.1 m yr^−1^ and channel widening of 0.12 ± 0.12 m yr^−1^ (Supplementary Table S6). Also, spatial analysis of the exposed roots suggests that the channel evolved during specific pulses rather than steadily through time (Supplementary Table S7 and Fig. S8). Our results also suggest that the widening started in the central part of the channel segment and then evolved rapidly due to earth falls generated by channel bed incision. As the channel widened the rate of widening decreased significantly.

**Figure 3:**
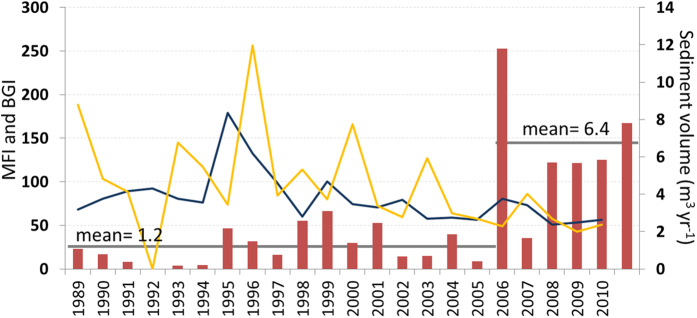
Estimated annual volume (m^3^) of sediment eroded for the period 1990–2012 (red bars), as well as the obtained Modified Fournier index (MFI: blue line) and the Bagnouls-Gaussen index (BGI *x-1*: orange line). Sediment yield data show a clear change in the trend after 2007, but are not correlated with high IF values.

The total eroded volume for the period 1989–2012 was computed to be 58.5 m^3^. From the channel reconstruction, the greatest erosion episode took place in 2007, when ~11.7 m^3^ of sand was mobilized in the system. The reconstruction also reveals a change in erosion rates after 2007. That is, the period 1989-2006 shows a mean erosion volume of 1.2 m^3^ yr^−1^, but has since become more than five times larger (6.4 m^3^ yr^−1^) (Fig. 3). These findings point to an intense, recent gully retreat (above the median retreat observed worldwide- 0.89 m yr^−1 ^30), although the catchment area that is delivering runoff and material to the channel is relatively small (~1.3 ha).

### Controls on gully evolution

The erosion-resistant lithology (i.e. gneiss) at the local base level of the drainage network has assured invariant base-level conditions at the study site over the course of the past several centuries. The documented rapid gully evolution and channel incision cannot therefore result from a lowering of the regional base level and thus must be related to different environmental causes. The long-term, annually resolved gully reconstruction presented here provides evidence to assess human-induced erosion processes and response to climate and vegetation cover changes in this unique landscape.

Our data and observations indicate an asynchronous response between sheet erosion, gully incision and vegetation changes. After the implementation of reforestation activities in the 1960–70s, vegetation cover inside this gullied slope increased by >70%. Our data demonstrate that this increment of vegetation cover reduced sheet erosion by almost 32% compared to HRUs without tree cover, an effect in agreement with previous studies^31^,^32^,^33^. However, this increase in vegetation cover was not able to prevent the initiation and evolution of the main gully channel through retreat and expansion. Field measurement suggest that the root diameter in the gully wall and surface level ranged between 16.3 ± 10.0 mm and 19.9 ± 12.7 mm, respectively, which provide average root density by 1.29 ± 0.48 cm^2^/m^2^ and 1.08 ± 0.44 cm^2^/m^2^ (Supplementary Table S9). Although the evolution of the gully was temporally controlled by the position of roots^29^,^34^,^35^ (see supplementary Table S7 and Fig. S8), the existence of mature trees with developed root systems was not sufficient to prevent the evolution of the gully. This finding reflects that different mechanisms of erosion drive sheet erosion and gully head and wall erosion, and suggests that adjustments to channel-slope connectivity had greater effect than the protective effect of roots on slopes.

In addition, our root-based reconstruction also documents changes in knickpoint retreat after 2007. The observed shift in channel incision trends was related to a short-lived, highly-localized and very intense downpour that took place on 25 May 2007^18^. Despite storm lightning damage compromising the rain gauge data and thus the precipitation records, fields measurements indicate a sediment yield of almost 44.1 tons for an area of 15 ha, or a specific sediment yield of 2.94 ton ha^−1^ (volume = 31.5 m^3^, specific density = 1.4 g cm^−3^, ref. 18), causing the disruption of the nearby road. Interestingly, we did not observe similar gully behavior after the subsequent intense rainstorm on September 9^th^, 2008 (69.4 mm in 173 min and 72.4mm h^−1^ in 30 min; ref. 36). Significant changes are also missing in 1996, which is considered one of the most erosive years in the Iberian Peninsula when a multitude of erosive processes were observed in other badlands in southern Spain^37^. Our analysis did not find significant correlations between gully retreat and climate variables^30^. Our analyses only found low correlations with the Bagnouls-Gaussen index (~0.3 for Spearman’s correlation coefficient, Fig. 3) pointing to a potential, but limited, role of the interactive effect of temperature and precipitation (Supplementary Fig. S10 and Table S11). Although we cannot ignore the role of hydro-repellence in the runoff due to pine exudates during warmer periods^38^, the unexpected behavior and lack of climate response is consistent with expectations for highly sensitive badlands in their initial evolutionary state^24^, as suggested from the historical perspective of this landscape.

Our results point to an asymmetrical response of the gully system to reforestation. Initially, the newly growing forest greatly reduced sheet erosion on the slopes^31^,^32^,^33^, but then concentrated erosion in the channel increased due to reduced sediment delivery from the slopes^39^. These phenomena might have resulted in decreased sediment connectivity between the slopes and the channel, which in combination with intense downpours would have favoured the occurrence of cut-and fill processes. These processes favoured channel capture and consequently major morphological changes of the drained system to re-adjust the equilibrium slope. Once headcut retreat was initiated in the badland system, its headward advance and lateral erosion will continue until the channel reaches a new equilibrium state (Supplementary Fig. S12). Morphological changes in badland systems have been hypothesized to reflect threshold exceedance^40^,^41^, and our findings support the idea that changes in connectivity between hillslopes and channels play an important role in badland evolution independent of climate effects^24^,^42^,^43^. This is especially true in semi-arid badlands that lack the restorative effect of perennial flows enhancing hillslope-channel connectivity, and where intense runoff events produce internal changes in the connectivity of different geomorphic elements that, in turn, condition the impact of subsequent events^24^. These findings support the interpretation that enhanced connectivity between hillslope and channel processes is a key consideration in the reclamation of disturbed lands.

## Conclusions and Implications for Landscape Restoration

Historical documents and accounts together with aerial photograph interpretation have provided new evidence of the anthropogenic origin and age of badlands in central Spain. Our findings suggest that these landscapes started to be intensively degraded after the 18^th^ century, becoming badlands with some of the largest observed degradation rates worldwide. The detailed, retrospective analysis of root exposure has allowed understanding of the recent past and the evolution of the Barranca de los Pinos gully, thereby providing new evidence about the role of reforestation practices in sensitive geomorphic environments. We argue that this information is relevant for informing reclamation of currently mined or quarried areas in comparable settings and that considering badland evolution state is necessary for proper evaluation of future reclamation practices.

Our results also show that reclamation measures based on reforestation alone may be a partial solution to sheet erosion control, but inadequate when it comes to gully control in severely unstable environments. Hence, shifting from vegetation-based to geomorphic-based recovery planning seems to be called for to assess the role of connectivity between processes in establishing a new equilibrium state, especially in the first stage of badland evolution. Vegetation recovery (as it is applied in many restoration projects), even if successful, may be a temporary, incomplete (and perhaps even counter-productive) solution, as channel erosion can be reactivated with unforeseen and counter-intuitive consequences.

## Methods

Historical archives referring to the economic evolution of nearby Pedraza town were consulted to search for direct descriptions of mining activity or about the badlands in general. Time series of aerial photography from the study site was available for the period 1946–2010; data was georeferenced using ArcGIS. We then digitized badland contours and interpreted major geomorphic features (i.e., channels and sediment deposits) as well as signs of anthropogenic activities related to mining.

Sheet erosion reconstructions were based on the analyses of 75 exposed roots taken in three different homogeneous units: i) HRU 1 is a poorly vegetated slope (~14° ± 5°) characterized by a low density of exposed roots; ii) HRU 2 is a steep (~26° ± 4°) forested interfluve characterized by a high density of transversal roots and dense pine needle cover; iii) HRU 3 is a bare interfluve with moderate slope (~16° ± 5°) and lack of vegetation cover or pine needles^25^,^26^. At the channel level, all the exposed roots’ sections were labelled, documented with photos and positioned with differential GPS (DGPS). Root density was assessed at both channel walls. To this end, we measured the diameter^31^,^32^,^33^ of each root in 24 plots of 0.5 × 0.5 m located at different channel sections along the gully. The topographical survey was performed using a DGPS (LEICA GPS 1200) to acquire the gully topography with centimetre precision (up to 4 points/m^2^). A chain handsaw was used to obtain the exposure root sections. Samples were stored in individual breathable bags and transported to be dried at room temperature. Sample preparation consisted of sanding and polishing with sand paper up to 400 grit. Then, tree rings were counted using a binocular microscope (Leica MS 5). The dating of tree-rings was carried out based on the comparative analysis of tree-width of 3 to 5 radii for each cross section. The recognition of the first year of exposure was defined by well-known anatomical criteria^25^,^26^,^44^,^45^.

Subsequently, a DEM of the gully channel was built from the points taken with GPS. 3D point data were imported into ArcGIS 9.3 and a TIN was generated using hard breaklines for the contour channel. The DEM thusly obtained had a resolution of 0.15 × 0.15 m, realistic for the existing density of points in the region of interest. We incorporated the position of each root as individual points and added the data relating to the dating of the exposure and roots sizes. The package ArcScene was used to analyse visually the spatial distribution of roots in the channel. For each root related with lateral erosion, a cross section was derived and orthogonal measurements were carried out between the position of the root and channel border. Additionally, the relative position of transversal roots upstream and in the vertical axis was also assessed. Due to the small secondary growth (average 8.5 mm) and its marginal impact on gully erosion estimation, this parameter was not taken into account in this study. Erosion rates were derived from dividing the relative distance between two roots and over the span between the time elapsed since the root was exposed. Erosion rates were then analysed for individual groups to extract channel retreat behaviour. The Supplementary Tables S6, S7 and Fig. S8 show numeric and graphic results from analysing the exposed roots in specific channels profiles. A specific root-density analysis was performed at the studied site. *Pinus pinaster* Ait. roots are distributed all over a very recent surficial deposit that covers the silica sand substrata, at the reach were the gully retreat (headward incision) has occurred. Prior to the gully incision, this surficial deposit had an average depth of 30.4 ± 6.4 cm (n = 6). Root density field measurements were carried out both in section-profile (at the walls of the gully) and in plan view on eight plots 0.5 × 0.5 m. In section, the average section of these roots is 16.3 ± 10.0 mm (n = 44). An average root density of 1.29 ±  = 0.48 cm^2^/m^2^ was obtained. In plan view, an average root density of 1.08 ± = 0.44 cm^2^/m^2^ was obtained. An average section of these roots is 19.9 ± 12.7 mm (n = 9) (Supplementary data). An interpretation about the behaviour from each group is also noted. From the data, it is clear that erosion is controlled by the root density. It is noticeable that the process starts in central position and then there is widening process. We used the Modified Fournier Index^46^ (Equation1) and the Bagnouls-Gaussen index^47^ (Equation2) to determine regional rainfall erosivity and aridity at the study site. Additionally, we also tested whether the average air temperature *T*_*m*_and the average precipitation in the wettest month *P*_*m*_were correlated with the gully retreat^30^. To this end, we used Spearman correlation^30^:


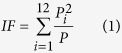


where *P*_*i*_ is the monthly average rainfall for month *i* (mm), and *P* is the average annual rainfall (mm). Kendall correlations were performed to determine the climate influence on sediment delivery:


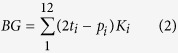


where *t*_*i*_ is the mean air temperature for month i, *P*_*i*_ is the total precipitation for month i in mm; and k represents the proportion of month during which 2 *t*_*i*_ − *P*_*i*_ > 0.

## Additional Information

**How to cite this article**: Ballesteros Cánovas, J. A. *et al*. Gully evolution and geomorphic adjustments of badlands to reforestation. *Sci. Rep.* **7**, 45027; doi: 10.1038/srep45027 (2017).

**Publisher's note:** Springer Nature remains neutral with regard to jurisdictional claims in published maps and institutional affiliations.

## Supplementary Material

Supplementary Information
