# Supplementary material for: Gully evolution and geomorphic adjustments of badlands to reforestation
Source: Sci Rep. 2017 Mar 22;7:45027. doi: 10.1038/srep45027 (PMC5361202; doi:10.1038/srep45027)
Supplement: Supplementary Information [file srep45027-s1.pdf]

Supplementary information

Gully evolution and geomorphic adjustments of badlands to reforestation

Ballesteros Cánovas J.A. (1); Stoffel M. (1,2); Martín-Duque J.F. (3); Corona C. (4); Lucía A. (5,6); Bodoque J.M. (7); Montgomery D. R. (8)

(1) Climatic Change and Climate Impacts, Institute for Environmental Sciences, University of Geneva, Boulevard Carl-Vogt 66, CH-1205 Geneva, Switzerland

(2) Department of Earth and Environmental Sciences, University of Geneva, CH-1205 Geneva, Switzerland

(3) Department of Geodynamics, Complutense University of Madrid (UCM) and IGEO (CSIC, UCM), E-28040, Madrid, Spain

(4) Centre National de Recherche Scientifique (CNRS) UMR6042 Geolab, F-63006, Clermont-Ferrand Cedex, France

(5) Faculty of Science and Technology, Free University of Bozen-Bolzano, I- 39100, Bolzano,Italy.

(6) Center of Applied Geosciences. Eberhard Karls University of Tübingen, G- 72074, Tübingen, Germany

(7) Department of Geological and Mining Engineering, University of Castilla-La Mancha (UCLM), Avda. Carlos III s/n, 45071 Toledo, Spain

(8) Department of Earth and Space Sciences. University of Washington, WA 98105, Seattle, USA

Corresponding author: Juan Antonio Ballesteros Canovas; (1) Climatic Change and Climate Impacts, Institute for Environmental Sciences, University of Geneva, CH-1205 Geneva, Switzerland. Telephone: +41(0)316318773. Email:juan.ballesteros@unige.ch

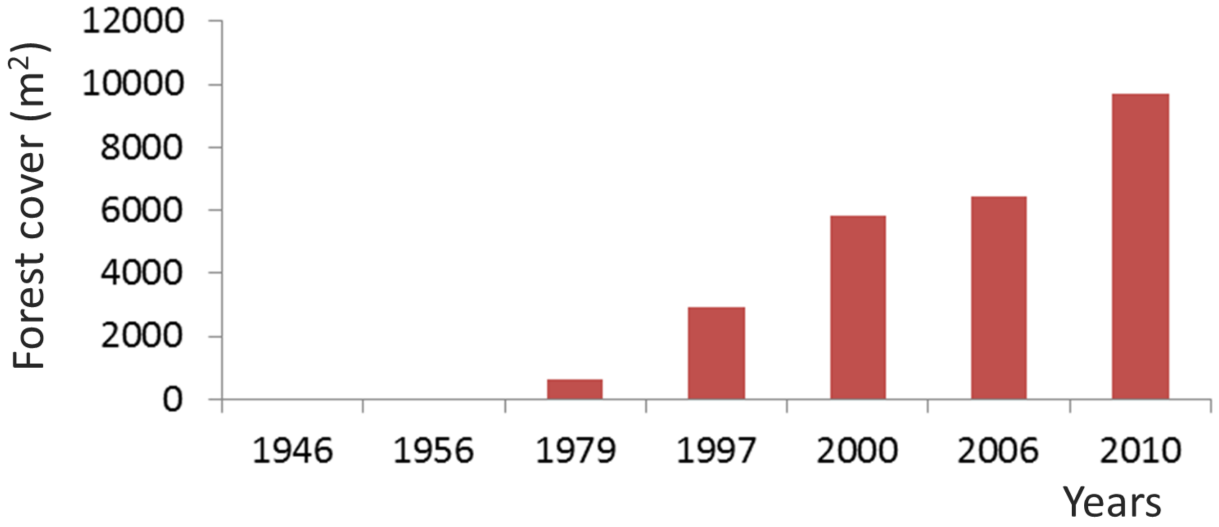

Supplementary Figure S1. Evolution of the forest cover within the gully system. Data based on aerial photograph-based recognition of forest cover in the studied gully. Forest cover first appears in late in 70's, suggesting that reforestation took place late 60's. The exact date is unknown and not available from the local forest service.

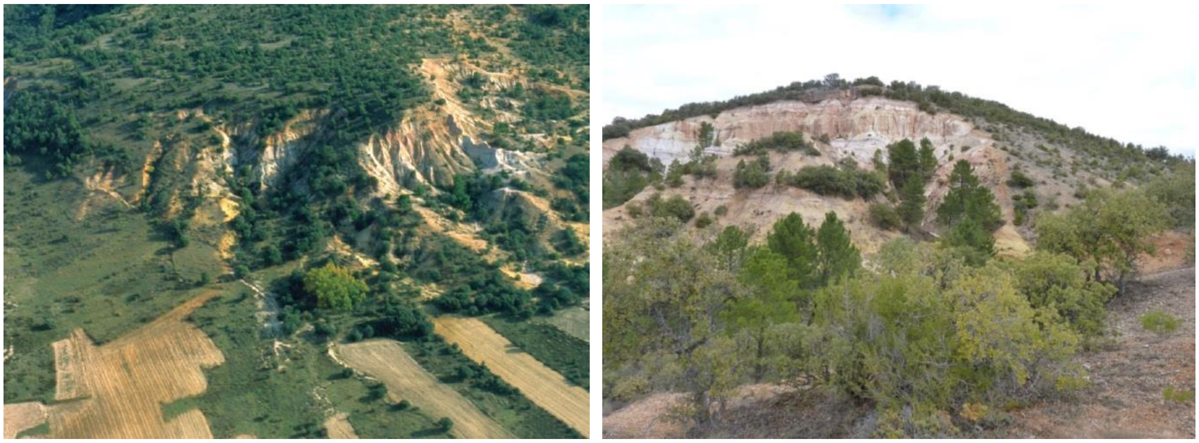

Supplementary Figure S2: Overview of the sandy badlands studied, characteristic of Mediterranean environments of Central Spain.

Supplementary Table S3: Historical facts and written sources related to the studied landscape

N°	Reference	Time	Information	Implication
**48**	Timoteo de Antonio (1936). Monografía de Pedraza de la Sierra. Madrid, 157p	1st-4th century	Hypothetical existence of Pedraza (Pedraca), as a fortified village during Roman Period, supported by several historians (i.e Ptolomeo (1562) and the existence of sigillata hispánica ceramic [49]. The first documentary information about the existence of Pedraza is during the Medieval Ages (5th century), when Pedraza is cited as "Petraza" in the Vow of San Millán of the year 934	Human-induced environmental changes. The Roman mining activity in the Iberian Peninsula has been widely studied, even in Segovia province. Moreover, it has been suggested that after S.V, the mechanism of roman glass production [50], as other services, were more localized at regional or local scale [51]. There are, thus, some facts indicating that the origin of sand outcrop could be related with Roman activities at the study site, specifically for glass production. However, this hypothesis has not been supported.
**52**	S- Diego de Colmenares. 1846. Historia de la insigne ciudad de Segovia y compendio de las Historias de Castilla. Ed. Baeza, E. Segovia. 453 p.	15th-16th century	Pag 297:<br><br>*“Acudió a la defensa el hermano de García Herrera, quien hubiera perecido a no socorrerle cierto picapedrero, que, con un martillo, destrozó al moro la cabeza.”*<br><br>***Translation:***<br><br>"The brother of Garcia Herrero came to his defence, but he would have perished had it not been for the help of a stone-cutter who destroyed the Moor’s head with a blow of his hammer”	Maximum splendor in Pedraza. Major re-buildings. Carved limestone denotes the date of the rebuilding of Pedraza castle in 1569 ([48], pag 108). Existence of stonemason activities in Pedraza. Use of local limestones for building. Increase in mining activities for supplying building material. Older construction are constructed with Upper Cretaceous marine (limestone and dolostone), which constitute the natural protection of silica sand sediment outcrop.

				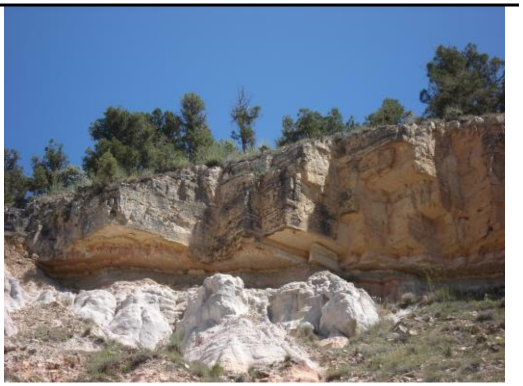<br>Marks of areas of old quarrying of carbonatic rocks (i.e. [53]), at the carbontatic cap of mesas and cuestas. Photo: Jose Francisco Martín-Duque.
**54**	Archive of Simancas. Book number 547, page 390	BC 18th century	From Timoteo de Antonio, 1936 pag:110<br><br>“Encuesta realizada por la Dirección general de Rentas<br><br>A mediados del siglo XVIII Pedraza, era cabeza de partido, conserva su importancia, reuniendo 150 vecinos de muros adentro, entre los cuales había propietarios que vivían de sus rentas, ganaderos importantes, seis sacerdotes, médico, cirujano, farmacéutico, un corregidor con sueldo, un abogado, un maestro, un preceptor de gramática, un curial romano, un administrador de tabacos y rentas generales, otro administrador de lanas, cinco administradores de haciendas de particulares, un arrendador de diezmos, un visitador de lanas, un fiel de romana, tres sacristanes, un abacero, tres tratantes de pescados y escabeches, cinco en ganado mular, un tratante en compro de terneras, doce comerciantes en lana fina, cuatro factores y un ayudante de ellas, diez y seis apartadores de lana, un sotamayoral de ganado lanar, dos mesoneros, dos taberneros, el que rige el reloj, un tablajero, un alguacil mayor,	Despite the importance of the village, the results of the survey indicate that there were not formal mining activities in 1751. At this time, neither sand nor limestone mining were considered an economic activity for the region. Major activities were agriculture and animal husbandry.

dos alguaciles ordinarios, catorce tejedores de lienzo, tres maestros sastres con su aprendices y oficiales, tres maestros de obra prima y cuatro de viejo, un cardador, un carpintero, dos peroiles, un cerero, un puerta ventanista, dos albañiles, dos herradores y un tallista con su oficial y aprendiz, sesenta y un labradores inclusos hijos y criados y veinte jornaleros, ocho pastores, diez pobres de solemnidad”

Translation:

Survey carried out by the Excise Authorities

In the mid-18th century, Pedraza was the county town and maintained its importance, bringing together some 150 citizens within the walls, including proprietors who lived from their rents, important cattle farmers, six priests, a doctor, a surgeon, an apothecary, an official magistrate, an advocate, a school master, a preceptor and grammarian, a representative of the roman curia, an administrator responsible for tobacco and general revenues, a wool administrator, five administrators of private estates, a tithe collector, a wool inspector, a slaughterhouse official, three sacristans, a grocer, three fish and pickle merchants, five mule traders, a calf dealer, twelve fine wool merchants, four factors and one of their assistants, sixteen wool sorters, an important sheep drover, two innkeepers, two tavern owners, the clockmaker, a butcher, a chief bailiff and two ordinary bailiffs, fourteen linen weavers, three master tailors with their apprentices and officials, three master shoemakers and four cobblers, a wool carder, a carpenter, a chandler, a door and window maker, two bricklayers, two blacksmiths, a wood carver with his skilled assistant and apprentice, sixty one farmers including their sons and servants and twenty day labourers, eight shepherds and ten paupers.

**21 & 55**	Morales-Molino et al., (2010)<br><br>Franco-Mugica et al., (2005)	18th – 20th centuries	Tree pollen analyses suggest that during the 'last millennium there have been several episodes of deforestation / recovery pinewood caused primarily by human activities. An intense deforestation was specially observed since 18th century until mid 20th century. These observation are supported by historical descriptions (19; 44)	Increase hillslope susceptibility to soil erosion. Driver of changes in economic activities.
**56**	Lozoya and Lopez de Ayala (1968)<br><br>Digital edition: Boletín de la Real Academia de Bellas Artes de San Fernando, núm. 26, p.101	18th century	Church dating 10th-12nd centuries, which was declared a historic-artistic monument in 1967, and later restored in 1969 because it had been buried by a debris cone from one of the gullies that make up the study area.<br><br>“La torre cae sobre el del Evangelio y de sus dos cuerpos, el superior no es el original al tener que rehacerse en 1756 por amenazar ruina”<br><br>**Translation:**<br><br>"The tower fell on the Gospel side and the upper of its two sections is not the original, as it was in danger of collapse in 1756 and had to be rebuilt."	See Figure S1-F<br><br>As the Church was rebuilt in 1756, the sand removed in 1969 had to be deposited between those times. Given that during second half 18th century the Church was an important ecclesiastic centre, it is plausible that the sand deposition can be constrained between 19th and mid 20th centuries.<br>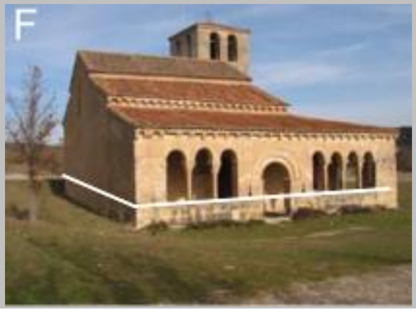
**57**	Eugenio Larruga (1791). Tomo XI	18th century	Page 1-2:<br><br>“Se hayan tierras muy buenas en estas provincias, como son: … y tres variedades de arena, la una de grano grueso ...y la tercera más menuda con la que se da pulimento a los cristales	First written evidence of:<br><br>1) Sand mining in the province, for glass factories<br><br>2) Existence of mining in the study

	“Memoria Politica y Económica sobre los frutos, comercio, fábrica y minas de España”		grandes”<br><br>**Translation:**<br><br>***"***Very good land is to be found in these provinces, such as: … and three kinds of sand, the first coarse-grained … and the third finer-grained which is used for polishing large glass."<br><br>Page 3.<br><br>En Pajares (2) y Rebollo, se ven una cantera blanca transparente<br><br>(2): Lugar del partido de Pedraza, es de Señorio y tiene 28 vecinos…<br><br>**Translation:**<br><br>***“In Pajares (2) and Rebollo, there is a quarry with transparent white stone.***<br><br>***(2): Pajares is located in nearby Pedraza, it is a Señorio and has 28 neighbours ...”***	area.
**58**	Rey de Viñas, P. (1994): Historia de la Real Fábrica de Cristales de San Ildefonso durante la época de la Ilustración (1720-1810). Madrid.	18th – 19th centuries	An important glass factory was developed at La Granja de San Ildefonso, placed 30 km away from the study site from mid 18th century. This factory was gaining notoriety to become the most important in Europe in the late 18th century. As the main raw material used silica sand collecting around Segovia. At the beginning of 19th century, a new glass factory was built in the village of Coca, located 53 km away the study site. The demand for sand and glass production of the two factories was decreasing until stop the factory of Coca due to the shortage of wood (intense deforestation) and national and international, including the Independence Spanish war. Since	It is plausible that the very rich silica sand could constitute a raw material for the Glass factory.

			mid 19th century until now the glass factory has continued.<br><br>Chemical analyses of glass from the first production suggest a very rich silica content (>70%). The existing documents suggest that the sand sources were located close Segovia and surroundings.	
**59**	Madoz (1849) Diccionario Geografico-Estadistico-Historico de España y sus posesiones de ultramar. Madrid, Tomo XII, 834 pp	early 19th century	This work provides detailed descriptions of all the municipalities of Spain corresponding to the first half of the nineteenth century. In many municipalities it has proven the existence of small glass furnaces and description of the mining and geomorphological activity; however in the main population centres in the study area we have not found clear evidence for the existence of glass factories or mining. We only found some description referring to stony terrain and sandstone terrain. Apparently, the description in this site indicate that the grass were good and productive at that time.<br><br>Orejana (PAG: 303 TOMOXII) sobre el Termino " se comprende varios montes de encina y enebro, en los que se críen muchas yerbas aromáticas, algún viñedo y diferentes prados de riego y secano , con buenos y abundantes pastos; pasa por el pueblo un arroyo que tiene su origen en las sierras carpetanas y desemboca en el r. Cega: el Terreno es de inferior calidad, pedregoso y arenisco."<br><br>PAJARES DE PEDRAZA (PAG: 517 TOMO XII): sobre el Termino "comprende 2 grandes montes de enebro, en los cuales se "críen muchas yerbas aromáticas; unos pequeños prados de siego, algún viñedo y diferentes praderas de buenos pastos-"<br><br>PEDRAZA (PAG: 734 TOMO XII): sobre el término "comprende un despoblado titulado Meteroso, un monte de encina y chaparro, bastante grande, en el queso críen yerbas	No clear description of sand mining activity in the main population centre located at the study area. However, we observed description of good grass for agriculture, and clear recognition of the nature of stone and sandstone terrain.

aromáticas, sirviendo de leñas para combustible; una buena dehesa con monte enebral, de excelentes pastos, titulada, Prado Monje, y bastantes prados de siega secano: pasan por este término el río Cega, y un arroyo titulado el Vadillo. El TERRENO en su mayor parte es poco feraz y de 3acalidad; teniendo una vega algo regular en el barranco de Velilla" Sobre la producción "P trigo, cebada, centeno, garbanzos, poco vino, hortalizas, leñas, pastos y lino; mantiene ganado lanar fino, cabrío, vacuno, asnal y mular; cría caza de liebres, conejos, perdices y otras aves, y pesca de barbos" sobre la industria "la agrícola, un buen lavadero de lanas, a 200 pasos del barrio de la Velilla, 2 batanes de sayales, 2 molinos harineros, 12 telares de lienzos, 3 de sayales y algunas tijeras de tundidores" Sobre el comercio " 2 tiendas de «eneros y comestibles, exportación de lanas y granos, é importación de los art. de que se carece en la v.: de tiempo inmemorial se celebra un mercado los martes de cada semana, en el que se presentan comestibles, granos, algunas tiendas de quincalla, ganados lanares, vacunos y de cerda, paños de Riaza y géneros de algodón"

Translation:

Orejana (Page: 303 Vol.XII) The District includes: various woodlands of holm oak and juniper with many aromatic herbs growing, some vines and various fields for both dryland and irrigated farming, and abundant good pastureland; flowing through the village there is a stream which rises in the Sierras Carpetanas and flows into the River Cega: the land is poor, stony and sandy.

PAJARES DE PEDRAZA (Page: 517 Vol. XII): This District includes: 2 large juniper woods where many aromatic herbs can be found; small fields for reaping, some vines and various fields

with good pasture.

PEDRAZA (Page: 734 Vol. XII): This District includes: an uninhabited area known as *Meteroso*, a large wood of holm oaks where aromatic herbs grow, and which are used for firewood; good pastureland with juniper woods and excellent pastures known as *Prado Monje*, and many dryland fields for reaping: the river Cega flows through this district and a stream known as el Vadillo. The land here for the most part is third class, with poor soil; there is a somewhat regular meadow in the steep Velilla valley. As to the produce: wheat, barley, rye, chickpeas, a little wine, vegetables, firewood, pastures and flax; for livestock: fine wool sheep, goats, cows, donkeys and mules; hares, rabbits, partridges and other fowl are caught, and there is fishing for barbel " ; the industries are farming, a good washing place for wool at 200 paces from the quarter of la Velilla, 2 fulling mills for homespun woollen cloth, 2 flour mills, 12 linen looms, 3 homespun wool looms and some trimmers' shears" As to commerce : 2 shops with goods and foodstuffs, exports of wool and grain and imports of any other articles which are lacking."

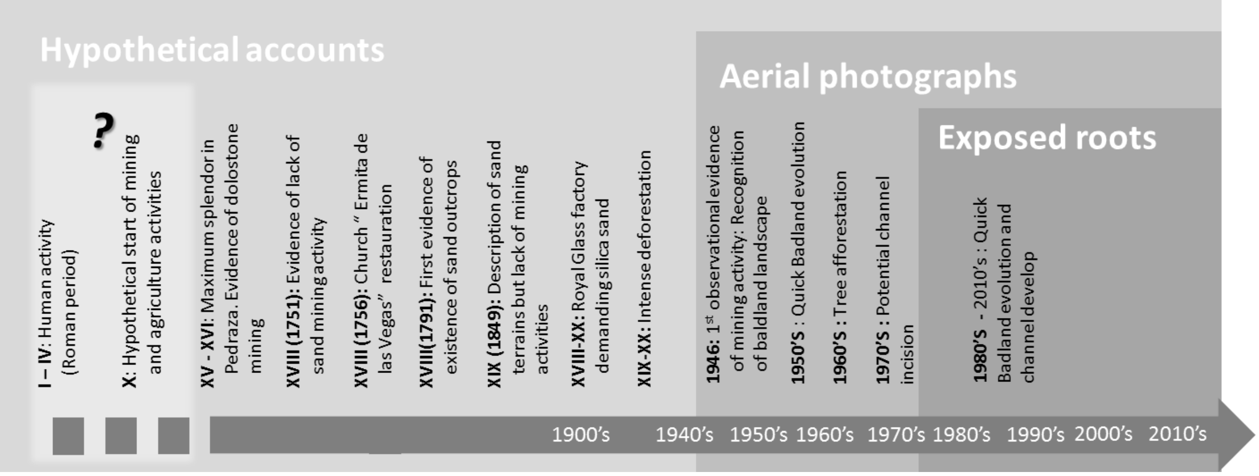

Supplementary Figure S4. Summary of the historical accounts and evidence to decipher the geomorphic behaviour of the studied Badlands.

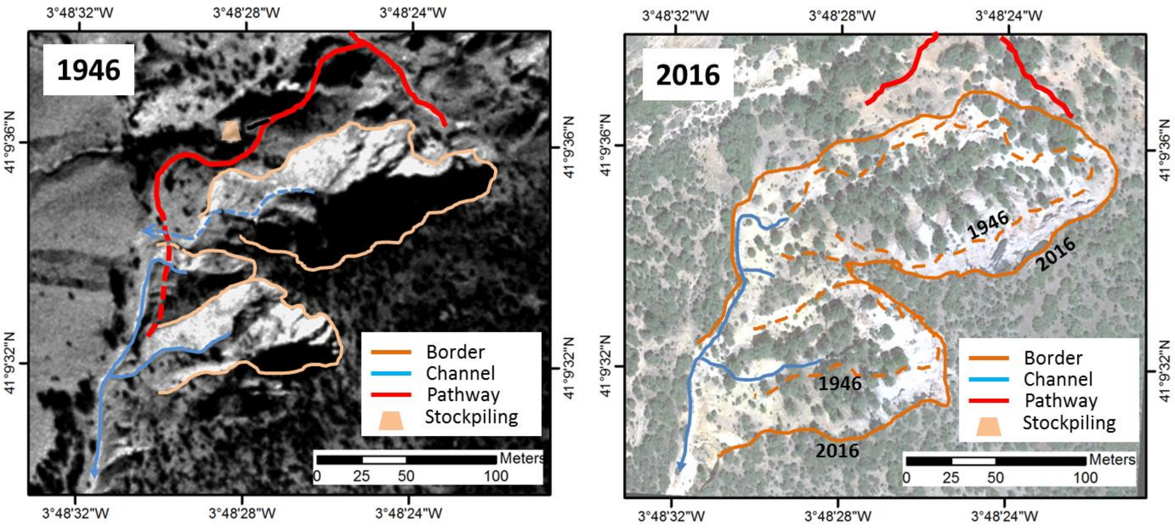

Supplementary Figure S5. Gully comparison between 1946 and 2016. Map has been created using ArcGIS 10.1 (www.esri.com). The aerial picture corresponds to U.S. Army flight in 1945-46 (http://fototeca.cnig.es/) (© Instituto Geográfico Nacional) and Google Earth V7.1.7.2606 (October 13, 2016) (http://www.earth.google.com)

Supplementary Table S6. Average annual amount of gully headcut advance and gully wall lateral retreat.

	Headcut advance (m)	Right retreat (m)	Left retreat (m)
**Average**	0.09	0.28	0.20
**Std.**	0.10	0.32	0.16
**Error**	0.01	0.06	0.04
**Range**	0.63	1.70	0.62
**Min**	0.01	0.00	0.00
**Max**	0.62	1.70	0.62
**N**	107.00	32.00	17.00
**ICL 95%**	**0.02**	0.12	0.08

Supplementary Table S7. Numerical results derived from analyzing the exposed roots at each channel profile.

Group	No. roots	Process	Phases	Observations/Interpretation
**GR1**	**6**	**Lateral channel widening**	P1: 0.5 m yr^{-1} (1994-1996)<br>$P1_R$: 0.7 m yr^{-1} (1994-1995)<br>$P2_L$: 0.014 m yr^{-1}(1996-2010)<br>$P2_R$: 0.03 m yr^{-1} (1995-2005)<br>$P3_L$: 0. 1 m yr^{-1} (2010-2012)<br>$P3_R$: 0.02 m yr^{-1} (2005-2012)	**Erosion starts in the central part of the channel** Substantial erosion in the first year, then stabilization, and finally moderate erosion rates
**GR2**	**10**	**Channel widening**	P1: 0.05 m yr^{-1} (2004-2005)<br>P2: 0.053 m yr^{-1} (2006-2012)	**Roots protect against progression of erosion** until 2006 when an earth fall exposes the entire group of roots at the same time
**GR3**	**12**	**Channel Incision; & longitudinal retreat**	P1: 0.083 m yr^{-1} (1996-1999)<br>P2: 0.03 m yr^{-1} (1999-2000)<br>P3: 0.01 m yr^{-1} (2000-2005)<br>P4: 0.148 m yr^{-1} (2005-2012)	**Incision is controlled by root density**. Low ratios match with high density root areas.
**GR4**	**3**	**Channel widening**	$P1_R$: 0.05 m yr^{-1} (2002-2011)<br>$P2_L$: 0.16 m yr^{-1} (2002-2005)<br>$P3_R$: 0.1 m yr^{-1} (2011-	**Erosion starts in the central part of the channel**

			2012) $P4_L$: 0.021 m yr^{-1} (2005-2012)	
**GR5**	**5**	**Channel Incision**	P1: 0.20 m yr^{-1} (1996-2000) P2: 0.01 m yr^{-1} (2000-2012)	The high erosion rate is explained by the absence of roots. This demonstrates that channel incision occurs as phases and is **controlled by the density of roots**
**GR6**	**7**	**Channel Incision**	P1: 0.373 m yr^{-1} (1996-1997) P2: 0.006 m yr^{-1} (1997-1999) P3: 0.09 m yr^{-1} (1999-2008) P4: 0.045 m yr^{-1} (2007-2012)	Two distinct phases of **incision processes** can be observed here, both are **controlled by root density**.
**GR7**	**6**	**Channel Incision**	P1: 0.005 m yr^{-1} (2000-2009) P2: 0.563 m yr^{-1} (2009) P3: 0.078 m yr^{-1} (2009-2012)	**Clear control of root density on erosion rates**. The large density of roots at the top results in a lower erosion rate; when the threshold is exceeded the rate increases until it decreases again when resistance emerges from deep roots
**GR8**	**6**	**Channel Incision**	P1: 0.01 m yr^{-1} (2002-2010) P2: 0.236 m yr^{-1} (2010-2011) P3: 0.134 m yr^{-1} (2011-2012)	Different phases suggest **an incision controlled by root density**.

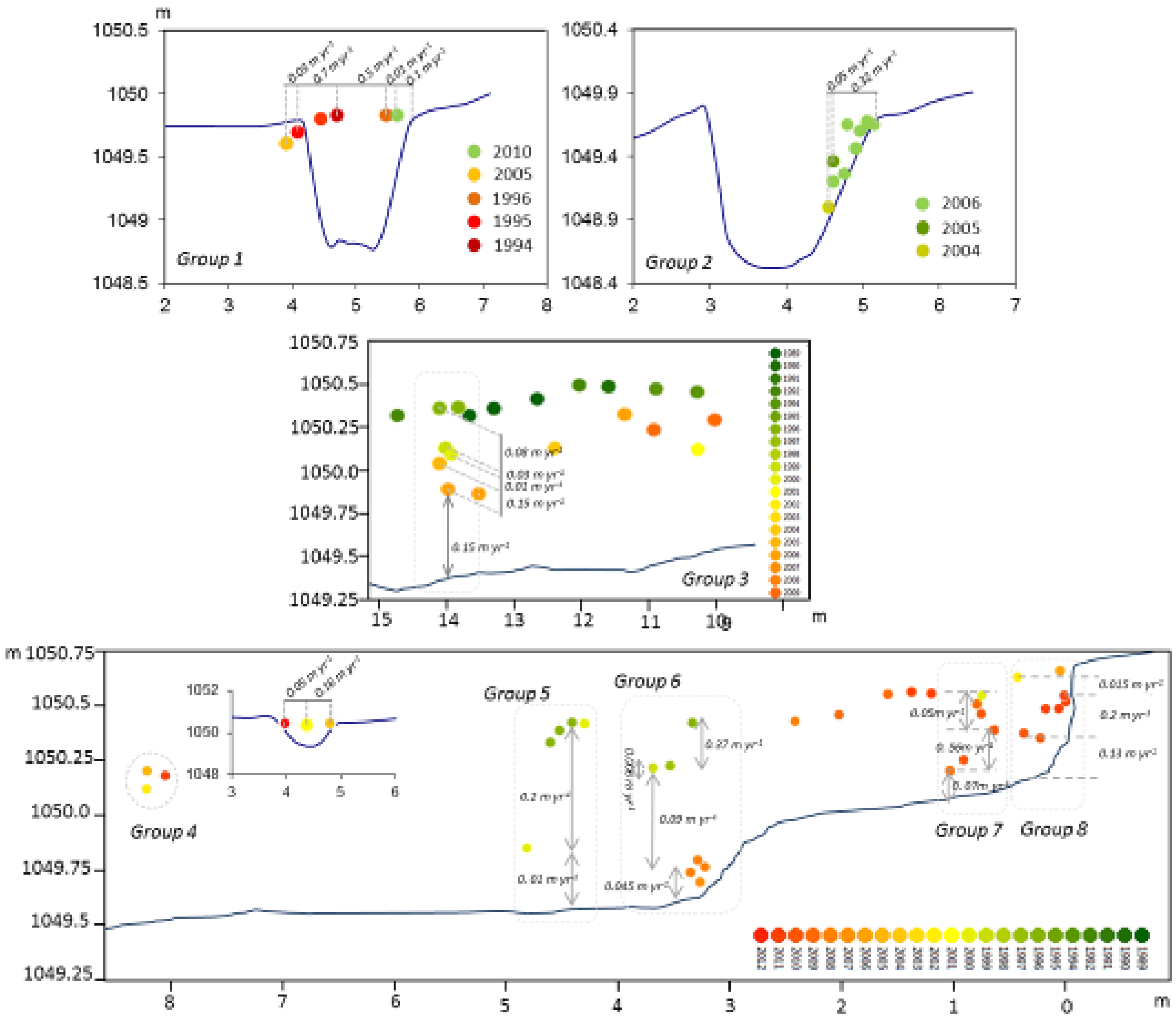

Supplementary Figure S8. Graphical view of exposed roots analyzed at each channel profile.

Supplementary Table S9. Measurement of root diameter and relative density at each plot 0.5x0.5 m. Plots 1 to 9 refer to the gully channel, whereas plot 10-12 refers to the surface area.

Plot	# Root	Diamter (mm)	Density root plot (%)	Plot	# Root	Diamter (mm)	Density root plot (%)	Plot	# Root	Diamter (mm)	Density root plot (%)
1	1	24	45	5	20	20.9	2	10	36	19.1	5
1	2	23	45	5	21	10.1	2	10	37	11.5	5
1	3	21	45	5	22	7	2	10	38	39.8	5
1	4	8	45	5	23	6.2	2	11	39	32.6	6
1	5	8	45	5	24	13.5	2	11	40	10	6
1	6	5.5	45	6	25	25.5	1	11	41	12.1	6
1	7	1.5	45	6	26	16.9	1	12	42	32.6	15
1	8	1.5	45	7	27	26.5	1	12	43	10	15
1	9	0.8	25	7	28	27	1	12	44	12.1	15
2	10	29	25	7	29	11	1				
2	11	22.5	25	8	30	43.1	1.5				
2	12	22	25	8	31	17.5	1.5				
2	13	10	25	8	32	13.8	1.5				
2	14	33	25	9	33	28	1				
2	15	12.5	33	9	34	15.2	1				
3	16	11.5	33	9	35	28	1				
3	17	9.8	33								
3	18	7.9	33								
3	19	9	33								

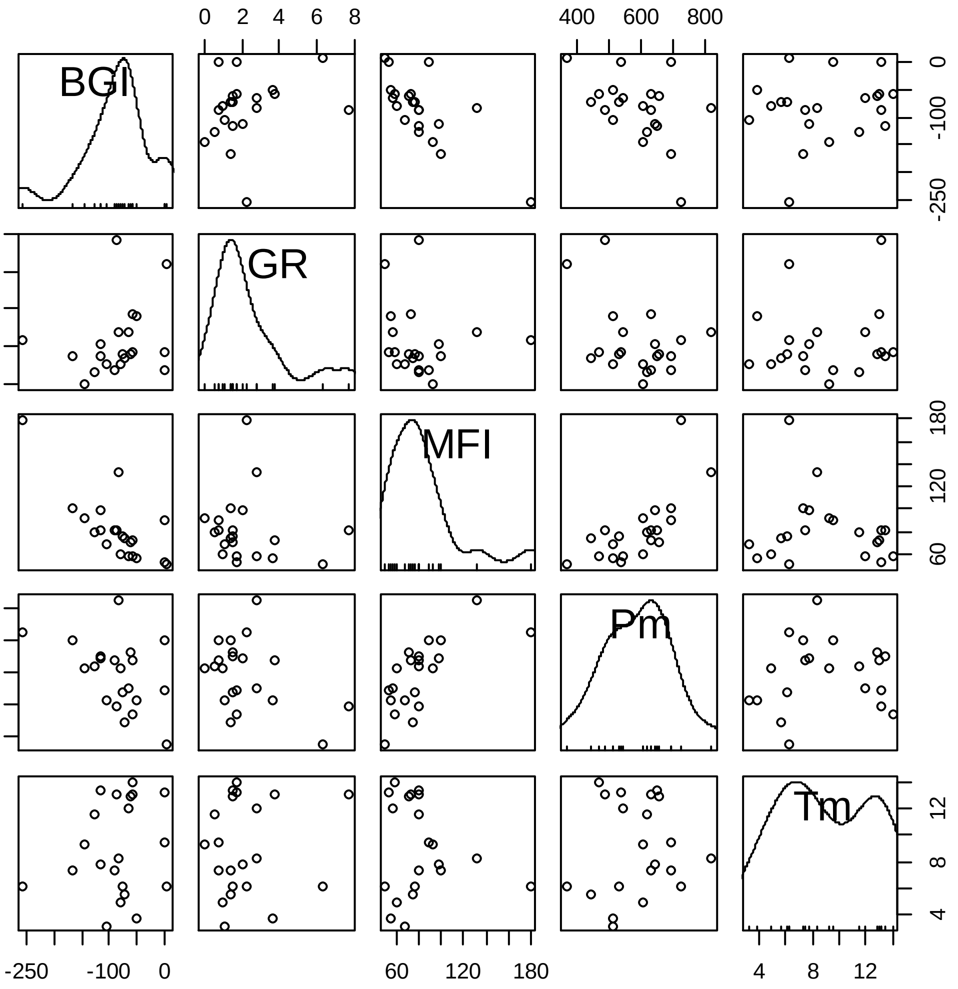

Supplementary Figure S10. Scatter plot matrix, where BG is the Bagnouls-Gaussen index, FI the Modified Fournier Index, GR is the gully retreat and, Pm is the precipitation of the wettest month and Tm is the average temperature (as per defined by Vanmaercke et al., 2016).

Supplementary Table S11. Spearman coefficient between gully retreat (GR) and climate variables.

	BGI	GR	MFI	Pm	Tm
BGI	1	0.37	-0.71	-0.42	0.13
GR	0.37	1	-0.21	-0.17	0.14
MFI	-0.71	-0.21	1	0.7	0
Pm	-0.42	-0.17	0.7	1	0.15
Tm	0.13	0.14	0	0.15	1

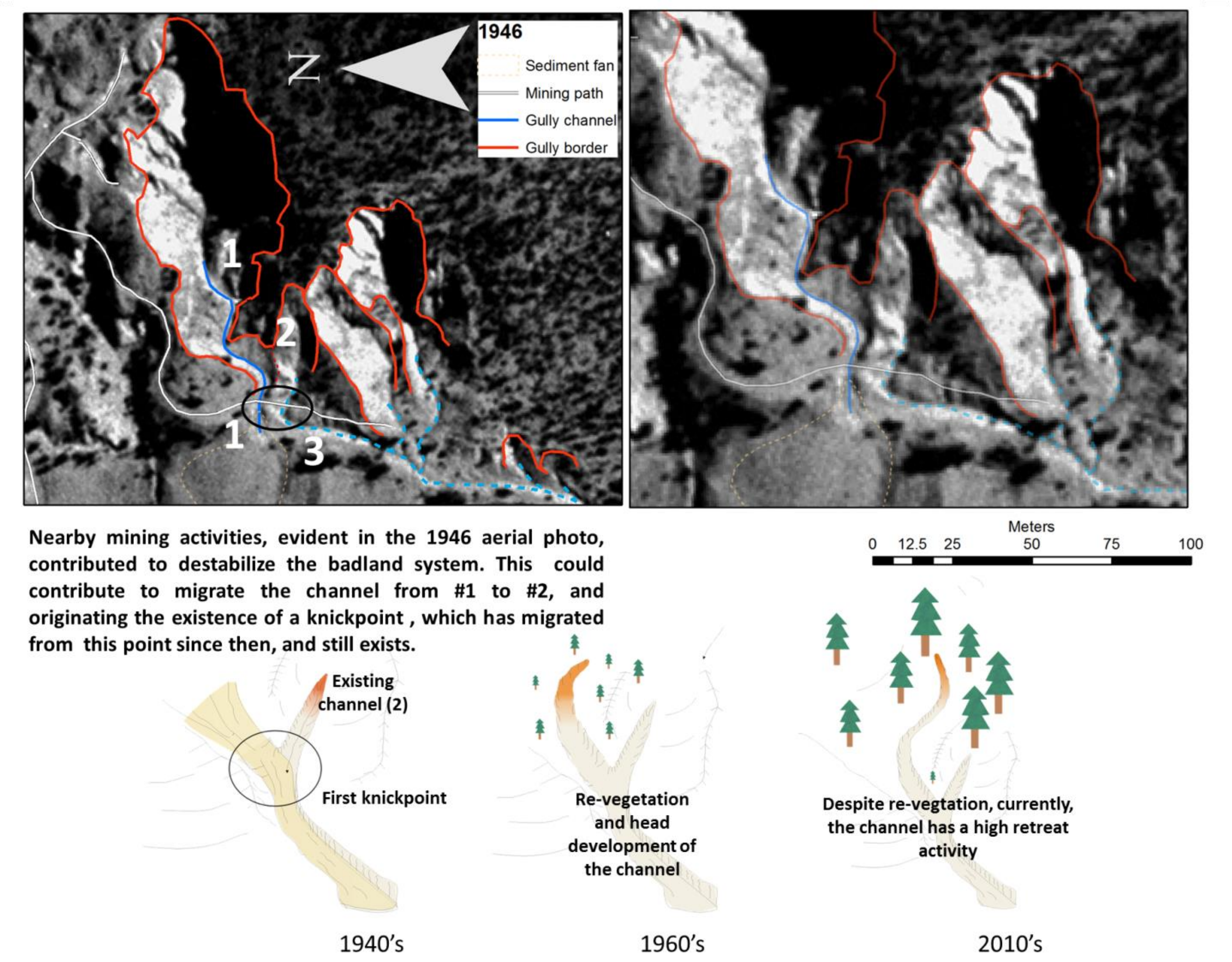

Supplementary Figure S12. Interpretation of geomorphic features related with the gully situation in 1946. The aerial photo interpretation and field recognition suggest that channels were independent for gullies (gullies #1 and #2). Field recognition could demonstrate the existence of a sedimentation fan located downstream from gully 1. Hypothetically, because the effect of an intense event, the channel from gully #2 may have captured the channel coming from gully #1 (point 3). The knickpoint was therefore transferred and evolved as a permanent feature of the current channel. Despite the afforestation and significant increase in the cover forest, currently it is possible to see high gully retreatment, which suggests a re-geomorphic adjustment, beyond the erosion stabilization provided by forest. Map has been created using ArcGIS 10.1 (www.esri.com). The aerial picture corresponds to U.S. Army flight in 1945-46 (http://fototeca.cnig.es/) (© Instituto Geográfico Nacional).
